# Dualistic Determinants of COVID-19 Vaccination Intention among University Students in China: From Perceived Personal Benefits to External Reasons of Perceived Social Benefits, Collectivism, and National Pride

**DOI:** 10.3390/vaccines9111323

**Published:** 2021-11-15

**Authors:** Phoenix K. H. Mo, Yanqiu Yu, Sitong Luo, Suhua Wang, Junfeng Zhao, Guohua Zhang, Lijuan Li, Liping Li, Joseph T. F. Lau

**Affiliations:** 1 Centre for Health Behaviours Research, Jockey Club School of Public Health and Primary Care, The Chinese University of Hong Kong, Hong Kong, China; phoenix.mo@cuhk.edu.hk (P.K.H.M.); yuyanqiu@link.cuhk.edu.hk (Y.Y.); 2 Vanke School of Public Health, Tsinghua University, Beijing 100084, China; sitongluo@mail.tsinghua.edu.cn; 3 Graduate School of Baotou Medical College, Baotou Medical College, Baotou 014040, China; bt_wangsuhua@163.com; 4 Department of Psychology, School of Education, Henan University, Kaifeng 475004, China; jfzhao63@hotmail.com; 5 Department of Psychology, School of Psychiatry, Wenzhou Medical University, Wenzhou 325035, China; zghcnu@wmu.edu.cn; 6 School of Public Health, Dali University, Dali 671003, China; lelejuan@126.com; 7 School of Public Health, Shantou University Medical College, Shantou 515063, China; lpli@stu.edu.cn

**Keywords:** vaccination intention, university students, perceived personal benefits, perceived social benefits, collectivism, national pride

## Abstract

Vaccination is one of the most effective ways of controlling the COVID-19 pandemic. However, vaccine hesitancy is prevalent, and relatively few studies have explored how variables related to personal and external motives have affected the intention to vaccinate. The present study investigated the association between perceived personal benefits, variables reflecting external motives (i.e., perceived social benefits, collectivism, and national pride) and intention to receive COVID-19 vaccination among university students in China. The interaction between perceived personal benefits and the three factors reflecting external motives on intention to receive COVID-19 vaccination was also examined. A total of 6922 university students from five provinces of China completed a cross-sectional survey. Results showed that adjusting for significant background variables, perceived personal benefits, perceived social benefits, collectivism, and national pride were all significant factors of intention to receive COVID-19 vaccination. Results from interaction analyses also showed that the association between perceived personal benefits and COVID-19 vaccination intention was stronger among those with lower levels of national pride. Findings highlighted the important role of self-directed interest and external motives in promoting uptake of COVID-19 vaccination.

## 1. Introduction

COVID-19 has caused significant morbidity and severe threats to public health across the globe. As of 2 August 2021, 120,837 COVID-19 cases have been reported in China, resulting in 5637 deaths [1]. Vaccination is recognized as the most successful and cost-effective public health interventions in reducing incidences and deaths of many infectious diseases [2]. Achieving a high coverage of vaccination at the population level is critical for protecting the community at large. One test-negative case-control real-world study on the effectiveness of inactivated COVID-19 vaccines against the Delta variant infection in China has shown that after adjusting for age and sex, the overall vaccine effectiveness for two-dose COVID-19 vaccination was 59.0% (95% confidence interval: 16.0% to 81.6%) against COVID-19, 70.2% (95% confidence interval: 29.6–89.3%) against moderate COVID-19, and 100% against severe COVID-19 [3]. Despite the ample evidence supporting the benefits of COVID-19 vaccination, vaccine hesitancy is prevalent in many countries [4,5]. For instance, a population-based survey among 8742 adults in China found that 35.5% of the participants reported COVID-19 vaccine hesitancy [6]. A recent review shows that only 68.4% of the global population is willing to receive COVID-19 vaccination [7], while a vaccination coverage of 55% to 82% of the population is needed to achieve COVID-19 herd immunity [8].

Previous studies investigating factors of COVID-19 vaccination intention primarily focused on socio-demographics (e.g., age, gender, education), cognitive factors (e.g., attitudes, perceived susceptibility, and perceived severity related to COVID-19) and psychosocial factors (e.g., personality, social support) [4,9,10]. Among them, perceived personal benefit and side effects are common ones [3,9,11]. According to the cost–reward analysis, people are motivated to behave in ways that maximize the rewards and minimize the costs [12]. COVID-19 vaccination yields personal benefits such as reducing the risk of infection, hospitalization and death. Perceived personal benefit is indeed a key construct of the Health Belief Model (HBM), which was associated with various health-related behaviors, including influenza vaccination [13], HPV vaccination [14], and COVID-19 vaccination intention [9,15].

In addition to the self-directed motive of perceived personal benefit, this study examined the associations between some external factors and COVID-19 vaccination intention, including the others-directed motive of perceived social benefits, and two other socio-cultural factors related to social capital (i.e., collectivism and national pride). COVID-19 vaccination can be viewed as a prosocial behavior, as it prevents transmissions to others and would contribute to herd immunity [16]. While previous studies sometimes suggest that self-benefit is the most important and sole motivator for receiving vaccinations [17], a growing number of studies have claimed that potential benefits to others and concerns of the others’ well-being are also important [18,19]. It is pivotal to understand whether individuals would take up COVID-19 vaccination purely out of their self-interest (personal benefits) or perceived social benefit, which is a less well studied potential factor of COVID-19 vaccination. Furthermore, the socio-ecological model suggests that structural factors (e.g., socio-cultural factors) are as important as individual-level factors in affecting health-related behaviors. Individuals’ cultural predispositions certainly contribute to the formation of their perceptions pertaining to vaccine benefits [20]. In particular, collectivism and national pride are socio-cultural components of social capital [21,22] that often enhance actions to improve community wellbeing [23]. Influenza vaccination [24] and use of protective measures to prevent COVID-19 [25,26,27] were associated with social capital. These two social capital external factors may thus affect COVID-19 vaccination independently from the effect of perceived personal benefits.

Collectivism was also tested in the present study as a potential factor of COVID-19 vaccination intention. A previous study found that collective responsibility was positively associated with COVID-19 vaccination intention [11]. In contrast to the individualists who tend to see themselves as unique and distinct from the group and value individual achievements, collectivists tend to conceptualize themselves within the context of their social surroundings and appreciate connectedness to others. Culturally upheld values concerning individualism versus collectivism underpin the level of prosocial tendencies. According to the empathy-altruism hypothesis, the sense of belonging to a common group evokes empathic arousal that promotes prosocial behaviors [28]. It is noteworthy that different countries vary along the spectrum of individualism versus collectivism. It is well documented that traditional Chinese culture emphasizes collective wellbeing while western countries tend to endorse individualism [29]. The understanding of the role of collectivism on COVID-19 vaccination in various countries may to some extent explain variations of COVID-19 vaccination rates across countries. There is a dearth of such studies.

The relationships between national pride and COVID-19 vaccination intention and behavior were also tested in the present study for the first time. Pride is an important positive emotion that plays a critical role in psychosocial well-being [30]. In general, the expression of pride indicates an individual’s success, social status, and group acceptance, and reinforces behaviors that generate the proud feelings and boost self-esteem. Feelings of pride can thus reinforce prosocial behaviors such as altruism and adaptive behaviors [31]. National pride would drive people to act collectively or even sacrifice to uphold national interest, especially at times of crisis [32]. Previous studies have found that national pride is related to prosocial behaviors [33]. COVID-19 is a serious national challenge. China has been under international pressure because of the pandemic. Those with stronger national pride may hence be more likely to take up vaccination to ‘protect’ the country and maintain their pride by ‘winning’ the battle against COVID-19 in the international setting.

Perceived personal self-benefits and the three ‘external factors’ (perceived social benefits, collectivism and national pride) are thus potentially independent, yet inter-related driving forces behind COVID-19 vaccination. Their relative importance and emphasis have strong bearing on health promotion strategies. No study has looked at the inter-relationships among these variables and COVID-19 vaccination intention. It is hypothesized that the associations between perceived social benefits/collectivism/national pride and COVID-19 vaccination intention would be independent from that between perceived personal benefit and vaccination intention. Thus, the significance between the three external factors and vaccination intention would become non-significant after controlling for perceived personal benefit. It is further hypothesized that, although individuals are primarily driven by perceived personal benefit, perceived personal benefit would be moderated by the three ‘external variables’ (i.e., perceived social benefits, collectivism, and national pride) in affecting vaccination intention. It is contended that the association between perceived personal benefits and vaccination intention might be weaker among those with high levels of perceived social benefits, collectivism, and national pride, as those people with high levels of ‘external factors’ might be less driven by self-interest when considering COVID-19 vaccination.

Given the background, the present study investigated factors of COVID-19 vaccination intention (given common mild side effects and 50%/80% vaccine efficacies in preventing COVID-19 infection, respectively) among university students in China. The studied factors included perceived personal benefits (i.e., internal motive), and three ‘external factors’ related to others-directed motive and social capital (i.e., perceived social benefits, collectivism and national pride). Second, it tested whether the three ‘external factors’ variables would be significantly associated with COVID-19 vaccination intention after adjusted for perceived personal benefit. Third, the interactions between perceived personal benefits and three ‘external motives’ variables were examined.

## 2. Materials and Methods

### 2.1. Participants and Data Collection

An anonymous cross-sectional survey was conducted during 1 to 28 November 2020 among university students in five Chinese provinces (Zhejiang (East China), Yunnan (Southeast), Guangdong (South), Inner Mongolia (North), and Henan (Central)) via an online survey link. A total of 165 classes of various grades (e.g., Year 1 to 4) mainly within the faculties of arts, sciences, social sciences, economics or management, engineering and medicine or pharmacy of the 5 participating universities were selected by convenience sampling. The collaborating teachers and student helpers sent an invitation message, the online survey link, and several reminders to all the students in the selected classes via WeChat groups that were being used for class administration. The inclusion criteria of participants included being a full-time student at the selected universities and able to read and write Chinese. The questionnaire was self-administered and took about 10–15 min to complete. It was written on the invitation message and the online questionnaire that the participation was anonymous, voluntary and confidential, and the return of the completed questionnaire implied informed consent. Upon completion, the participants could join a lottery draw which offered eight prizes of 10–50 RMB (about 1.5–7.5 USD) and a symbolic “lucky money” of 1 RMB for half of the participants in each participating university. Ethical approval was obtained from the ethics committee of the corresponding author’s affiliated association.

A total of 9593 invitations were sent out; 6940 students returned the completed questionnaires (a response rate of 72.3%), 18 of which were excluded due to incomplete data. Thus, 6922 participants were included in the final data analysis.

### 2.2. Measurements

#### 2.2.1. Background Characteristics

Background information was collected, including the studied province, gender, ethnicity, faculty, and grade. Their perceived risk of COVID-19 was measured using a single item “If not taking up COVID-19 vaccination, what is the chance that you would contract COVID-19 in the future one year? (1 = extremely low to 5 = extremely high)”.

#### 2.2.2. Behavioral Intention of COVID-19 Vaccination

Two items were used to examine behavioral intention of COVID-19 vaccination. Participants were asked to rate their chance of taking up free COVID-19 vaccination with common mild side effects and 80% and 50% efficacy in preventing COVID-19 infection, respectively, within the first six months upon the vaccines’ availability (likely/definitely yes versus likely/definitely not).

#### 2.2.3. Perceived Personal Benefits of COVID-19 Vaccination

Three items assessed the level of agreement with the following personal benefits in taking up COVID-19 vaccination: (i) self-protection, (ii) less frequent facemask wearing, and (iii) restoration of normal life. The items were rated on a 5-point Likert Scale from 1 = strongly disagree to 5 = strongly agree. The Cronbach’s alpha was 0.71 in this study.

#### 2.2.4. Perceived Social Benefits of COVID-19 Vaccination

Two item assessed participants’ level of agreement with the following social benefits of taking up COVID-19 vaccination: (i) effectively protect others from contracting COVID-19 via contacting you, and (ii) help the country to contain the COVID-19 pandemic and prevent another round of COVID-19 outbreak in China. The items were rated on a 5-point Likert Scale from 1 = strongly disagree to 5 = strongly agree. The Cronbach’s alpha was 0.80 in this study.

#### 2.2.5. Collectivism

Collectivism was assessed by the collectivism subscale of the 26-item Five-Dimensional Scale of Individual Cultural Values, which has been validated in among U.S. and Korean university students [34]. Two independent bilingual researchers translated the English version into Chinese, and the final Chinese version was approved by the corresponding author in this study. Sample items are “Individuals should sacrifice self-interest for the group” and “Group welfare is more important than individual rewards”. The items were rated on a 5-point Likert Scale ranging from 1 = strongly disagree to 5 = strongly agree; higher scores indicated higher levels of collectivism. The Cronbach’s alpha was 0.90 in this study.

#### 2.2.6. National Pride

National pride was assessed by using the four items modified from the 2013 ISSP (International Social Survey Programme) National Identity III questionnaire, which has been used in 33 countries [35] and applied to the Chinese population [36]. The four items were (i) “How proud are you of being a Chinese?” (1 = not proud at all to 4 = very proud), (ii) “How close do you feel to China?” (1 = not close at all to 4 = very close), (iii) “The world would be a better place if people from other countries were more like China” (1 = strongly disagree to 5 = strongly agree), and (iv) “Generally speaking, China is a better country than most other countries” (1 = strongly disagree to 5 = strongly agree). The Cronbach’s alpha was 0.75 in this study.

### 2.3. Statistical Analysis

The four independent variables (perceived personal benefits, perceived social benefits, collectivism, and national pride) were standardized to facilitate the comparisons of effect size in the subsequent analysis. Univariate and multivariate (with adjustment of background factors) logistic regression analysis was conducted to test the individual associations between the four independent variables and the two dependent variables (behavioral intention of free COVID-19 vaccination of mild side effects and 80% or 50% efficacy). Crude odds ratios (ORc) and adjusted odds ratios (ORa) and their respective 95% confidence intervals (CIs) were derived. Moderation analyses were conducted to test the significance of the interaction effects of the three interaction terms (perceived personal benefits × perceived social benefits; perceived personal benefits × collectivism, perceived personal benefits × national pride) on the two outcomes, after adjusting for background factors. Data analysis was conducted by using SPSS 21.0 (e.g., process macro for moderation analysis and binary logistic regression module for logistic regression analysis). Statistical significance was defined as two-tailed *p*-value < 0.05.

## 3. Results

### 3.1. Descriptive Statistics

#### 3.1.1. Background Characteristics

Of all the participants, about 40% were male (36.4%) and first-year students (43.2%). Half were studying medicine and health subjects (50.9%). The majority were Han ethnic (86.8%). About one-tenth (9.0%) perceived high risk of contracting COVID-19 in the future one year if not taking up COVID-19 vaccination (see Table 1).

#### 3.1.2. Behavioral Intention of COVID-19 Vaccination

Of the participants, 37.3% showed behavioral intention of receiving free COVID-19 vaccination of 80% efficacy and common mild effects within the first six months upon vaccines’ availability. The prevalence dropped to 19.8% if the efficacy were only 50% (see Table 1).

### 3.2. Factors of Behavioral Intention of COVID-19 Vaccination

Those of two particular provinces (e.g., Inner Mongolia and Yunnan), male sex, non-Han ethnicity, major in medicine and self-perceived high/extremely high risk of contracting COVID-19 were positively associated with behavioral intention of receiving free COVD-19 vaccination with common mild side effects and 80% and 50% efficacy. The associations between grade and the two outcomes were non-significant (see Table 2).

In Table 3, adjusted for the aforementioned significant background factors, separate logistic regression models show that perceived personal benefits (ORa = 1.36, 95% CI: 1.29–1.43), perceived social benefits (ORa = 1.44, 95% CI: 1.36–1.52), collectivism (ORa = 1.27, 95% CI: 1.20–1.33), and national pride (ORa = 1.15, 95% CI: 1.09–1.21) were all positively associated with intention of receiving free COVID-19 vaccination with common mild side effect and of 80% efficacy. The results were similar to those of another outcome of intention to receive COVID-19 vaccination involving 50% efficacy.

### 3.3. Interaction Analysis

In Model 1a,c,e of Table 4, it is seen that perceived social benefits, collectivism, and national pride were all statistically associated with vaccination intention after controlling for perceived personal benefits, if the COVID-19 vaccines were 80% efficacious and mild side effects are common, i.e., the external factors of perceived social benefits/collectivism/national pride and the personal motive were independently associated with COVID-19 vaccination intention under this circumstance. Similar analysis showed that collectivism, but not perceived social benefits and national pride, remained significantly associated with vaccination intention if the vaccines were 50% efficacious (Model 2a,c,e).

**Figure 1:**
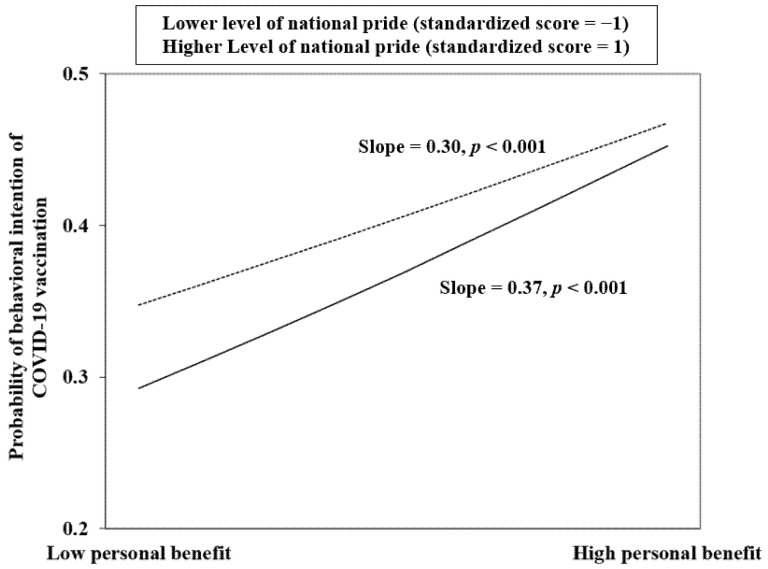
Graphical representation of significant interaction effect of perceived personal benefits × National pride onto behavioral intention of COVID-19 vaccination.

In Model 1b,d,f of Table 4, the interaction terms (i.e., perceived personal benefit × perceived social benefit, perceived personal benefits × collectivism, perceived personal benefits × national pride) were added to three aforementioned individual models (1a,c,e) that contain the two respective main effects. One of the three interaction models (perceived personal benefits × national pride for the dependent variable of vaccination intention given common mild side effects and 80% efficacy) was statistically significant. Such significant interaction effect was graphically demonstrated in Figure 1. It is seen that the association between perceived personal benefits and COVID-19 vaccination intention was stronger among those with lower levels of national pride (standardized score = −1; slope = 0.37; *p* < 0.001) than among those with higher levels (standardized score = 1; slope = 0.30; *p* < 0.001).

## 4. Discussion

To summarize, the study found prevalence of COVID-19 vaccination intention of about 37% if the efficacy of the vaccine were 80%, and as expected, dropped to about 19.8% if the efficacy was only 50%. The eventual vaccination rate may differ as health promotion, incentives, and policies would be introduced. As females, non-Hans (minorities), and those perceived lower risk of infection showed lower intention, health promotion may need to pay more attention to these subgroups. As seen from this study, the ‘external factors’ such as perceived social benefits, collectivism, and national pride were significantly associated with vaccination intention after adjusted for the background variables. Furthermore, the ‘external factors’ variables remained statistically significant in the 80% vaccine efficacy scenario after controlled for perceived personal benefits and the background variables. In addition, perceived personal benefits interacted significantly with national pride, i.e., the association between personal benefits and vaccination intention was stronger among those with lower national pride in the 80% efficacy scenario.

In the present study, corroborating extant literature [9,15], perceived personal benefits was positively associated with COVID-19 vaccination intention. This is understandable as individuals consistently weigh the relative benefits and harms of COVID-19 vaccination, and would decide to vaccinate when perception of benefits outweigh the harms. The primary benefit would be protection from COVID-19. As COVID-19 is perceived to have severe negative impacts to health and life in general, perceived benefit would certainly be one of the key factors affecting vaccination intention. Thus, it is doubtless that health promotion should enhance perceived benefit of COVID-19 vaccination, as such an approach has strong theoretical (e.g., Health Belief Model) and empirical support [9,15,37]. Further support of the personal benefit approach also comes from the results showing that perceived personal benefit remained significant in models that also include the three ‘external factors’; the effect size was also relatively large when compared with other ‘external motive’ variables.

Nevertheless, at the time of a social crisis such as the COVID-19 pandemic, prosocial behaviors have emerged and short-term self-interest is often sacrificed for longer-term collective interests [38]. Health promotion emphasizing on both personal benefits and the ‘external factors’ that was not directly related to self-interest, such as promoting perceived social benefits and appeals to collectivism and national pride, may be more effective than focusing on perceived personal benefits alone in improving the COVID-19 vaccination rate. Such dualistic approaches are justified as the ‘external factors’ showed independent associations with vaccination intention after adjusting for perceived personal benefits given 80% vaccine efficacy, i.e., the dualistic approach might have additional effect on COVID-19 vaccination intention. It is, however, interesting that perceived social benefits and national pride became ‘non-significant’ after adjusting for personal benefits given 50% efficacy while collectivism remained significantly associated with vaccination intention. It seems that at lower vaccine efficacies, the belief whether the vaccines could benefit oneself (e.g., protection from infection) would be the primary concern, and that the dualistic approach involving perceived social benefits/national pride might work better under circumstances of relatively high vaccine efficacy. Thus, future studies should confirm such associations and if validated, health promotion of COVID-19 vaccination may use strategies that emphasize perceived social benefits, collectivism, and national pride, especially if the available COVID-19 vaccine has relatively high efficacy.

This is the first study reporting a significant association between collectivism and COVID-19 vaccination. There are some plausible explanations. First, collectivists may tend to take the interest of others into account, they would be more likely to cooperate and show concerns with collective actions that would benefit to the group (COVID-19 vaccination in this case). Second, as they are more sensitive to others’ views, they are more likely to observe and adopt the social norm about a behavior, which is an important factor of behavioral intention and performance [39,40]. In the context of COVID-19, previous studies showed that collectivists tended to show more support and report higher descriptive and injunctive norms on COVID-19 preventive behaviors such as engaging in social distancing behavior and wearing mask [26], and were more likely than others to comply with social distancing and hygiene practices to help reduce the spread of COVID-19 [25]. Collectivists may be more responsive to descriptive norms and subjective norms [26], leading to a higher intention to receive COVID-19 vaccination.

Another novel finding was the association between national pride and COVID-19 vaccination intention after adjusting for the background factors and personal perceived benefits (given 80% vaccine efficacy). Dorfman et al. [41] found that the consideration of pride led to more cooperation in social dilemmas, of which the COVID-19 pandemic is a good example. National pride is a form of collective pride, defined as a sense of superiority experienced by a group of individuals due to the achievements of their in-group over those of out-groups [32]. According to the social identity theory, the groups of which people belonged to were important sources of pride [42]; nation is certainly one of the most important groups and their source of pride. In addition, as described in the Intergroup Emotions Theory [42], individuals experience positive emotions, e.g., pride, when they feel connected in a group to which they belong and with which they identify, such positive emotion increase one’s prosocial tendency towards individuals within the group. Furthermore, national pride is a positive emotion [22]. According to the Broaden and Build Theory, positive emotions can help to build up resources by producing a state of “social broadening” [43], which further promotes positive prosocial behaviors. Studies have found that pride at the collective level is related to prosocial behavior [33].

Interestingly, the present study observes that the association between perceived personal benefits and COVID-19 vaccination intention would be stronger among those with low level of national pride. It seems that the decision to receive COVID-19 vaccination among those with lower national pride are more driven by the self-interest of perceived benefits of COVID-19 vaccination. However, the interactions between perceived personal benefits and perceived social benefits/collectivism were non-significant. The understanding of the inter-relationships between perceived personal benefits and the ‘external motives’ variables are thus only preliminary and requires future investigations.

Our findings yield important theoretical and practical implications. First, it highlights the importance of emphasizing both personal and social benefits of COVID-19 vaccination when promoting COVID-19 vaccination intention. Messages that depict both the efficacy of vaccinations in protecting individuals from infection and promoting individual health and well-being and the benefits of herd immunity for other people (e.g., if you get vaccinated, then you would protect others who are not vaccinated) may be more effective than those mentioning personal benefits alone. In the current case of COVID-19 of which reaching herd immunity thresholds is crucial for controlling the pandemic, it may be particularly important to promote the awareness of the social benefits that vaccination is not only an individual but also a collective effort. Findings of the current study also provide promising evidence that collectivism contribute to higher intention to receive COVID-19 vaccination that may bring large benefits to the community. The current study extends previous work and highlights that collectivism would also contribute to intention to receive COVID-19 vaccination. To promote the uptake of COVID-19 vaccination, there is a need to promote a collectivistic orientation among the general population. Messages that emphasize social harmony, interpersonal relationship, and the importance of meeting social obligations and common goal can be useful techniques to encourage the population to receive COVID-19 vaccination. Future studies comparing levels of collectivism and national pride and COVID-19 vaccination coverage may test the contention that variations in such social capital would partially explain differences in national coverage rates.

The study involved a large sample and was timely. It, however, had several limitations. First, the study was cross-sectional in nature so causality could not be assumed. Data was collected from five provinces, and the sample contained a relatively large number of first year students and those from the medicine major. Therefore, findings may not be representative to the whole university student population in China. Participants were self-selected so the data might have been subject to self-selection bias. As COVID-19 vaccination was not yet available during the time of the study, participants were asked to rate their intention to receive COVID-19 vaccination within six months upon its availability. The situations (e.g., global epidemic, variants, evidence about efficacy and side effects, and policies) keep changing so that the obtained information on vaccination intention might be different from those of actual performance of vaccination. University students may also be very different from the general population and generalization may be limited. Future studies should include those with lower socioeconomic status, such as older populations, those living in rural areas, or individuals with a lower level of education and test the applicability of perceived benefits, collectivism, and national pride in these populations. Furthermore, some scales were developed for this study as they were not available in the literature, and other potential factors, such as perceived susceptibility and perceived severity of COVID, and history of COVID-19 infection, have not been investigated. University students may have a lower level of perceived susceptibility and severity of COVID-19 due to their relatively young age. The role of these factors on their vaccination intention might be different compared to the older populations or those with chronic diseases. Last, clustering within the schools have not been taken into account; multi-level analysis or meta-analytic approach might improve accuracy

## 5. Conclusions

It is doubtless that the success in reducing COVID-19 transmissions relies on people voluntarily adopting vaccination. Identifying the factors that may potentially promote intention to receive COVID-19 would allow health care professionals to develop evidence-based strategies to increase national COVID-19 vaccination coverages. Overall, the present study provides encouraging insights that emphasizing both individual and social benefits, and collectivism and national pride may be effective at increasing COVID-19 vaccination, therefore reducing the burden of the disease. If confirmed by future studies, tailored messages should be designed based on individual’s perceived benefits in order to maximize the effects of health interventions. The understanding of the roles of self-directed interest versus others-directed interest, related personal traits and social capitals (e.g., collectivism versus individualism) is an important possible new direction for research and health promotion of COVID-19 vaccination.

## Figures and Tables

**Table 1:** Descriptive statistics.

Background Factors	n/Mean	%/SD
Studied province	2597	37.5
Inner Mongolia	1943	28.1
Henan	931	13.4
Zhejiang	896	12.9
Yunnan	555	8.0
Guangdong		
Gender		
Female	4402	63.6
Male	2520	36.4
Ethnicity		
Else	913	13.2
Han	6009	86.8
Major		
Art; Social science; Economics and management	1637	23.6
Science; Engineering	1522	22.0
Medicine	3525	50.9
Others	238	3.4
Grade		
First year	2993	43.2
Second year	1894	27.4
Third year	1164	16.8
Fourth/fifth year	776	11.2
Postgraduate	95	1.4
Perceived risk		
Low-to-moderate	6298	91.0
High	624	9.0
**Behavioral Intention of COVID-19 Vaccination**		
80% efficacy + common mild side effects + free		
Definitely/likely not	4341	62.7
Likely/definitely yes	2581	37.3
50% efficacy + common mild side effects + free		
Definitely/likely not	5551	80.2
Likely/definitely yes	1371	19.8

**Table 2:** Background factors of behavioral intention of COVID-19 vaccination.

Background Factors	Behavioral Intention of Receiving Free COVID-19 Vaccination with Mild Side Effects	
	80% Efficacy	50% Efficacy
	ORc (95% CI)	ORc (95% CI)
Studied province		
Guangdong	Ref = 1.0	Ref = 1.0
Inner Mongolia	1.48 (1.21–1.80) ***	1.93 (1.50–2.49) ***
Henan	1.16 (0.95–1.43)	1.10 (0.84–1.44)
Zhejiang	1.18 (0.95–1.48)	1.27 (0.95–1.70)
Yunnan	1.66 (1.33–2.08) ***	1.73 (1.30–2.30) ***
Gender		
Female	Ref = 1.0	Ref = 1.0
Male	1.31 (1.18–1.45) ***	1.36 (1.21–1.53) ***
Ethnicity		
Han	Ref = 1.0	Ref = 1.0
Else	1.31 (1.13–1.50) ***	1.30 (1.10–1.54) **
Faculty		
Art; Social science; Economics and management	Ref = 1.0	Ref = 1.0
Science; Engineering	1.05 (0.91–1.22)	0.96 (0.80–1.15)
Medicine	1.17 (1.03–1.32) *	1.24 (1.06–1.43) **
Others	1.01 (0.76–1.34)	1.08 (0.76–1.52)
Grade		
Postgraduate	Ref = 1.0	Ref = 1.0
Fourth/fifth year	0.84 (0.54–1.31)	0.97 (0.55–1.71)
Third year	0.93 (0.61–1.44)	1.09 (0.62–1.90)
Second year	0.98 (0.64–1.50)	1.28 (0.74–2.22)
First year	1.02 (0.67–1.56)	1.31 (0.76–2.26)
Perceived risk		
Low-to-moderate	Ref = 1.0	Ref = 1.0
High	2.52 (2.13–2.98) ***	3.21 (2.70–3.81) ***

Note: ORc = crude odds ratio; CI = confidence interval; Ref = reference group. * *p* < 0.05; ** *p* < 0.01; *** *p* < 0.001.

**Table 3:** Individual associations between the independent variables and the two outcomes of behavioral intention of COVID-19 vaccination.

	Behavioral Intention of Receiving Free COVID-19 Vaccination with Common Mild Side Effects			
	80% Efficacy		50% Efficacy	
	ORc (95% CI)	ORa (95% CI)	ORc (95% CI)	ORa (95% CI)
Perceived personal benefits	1.42(1.34–1.49) ***	1.36(1.29–1.43) ***	1.45(1.36–1.54) ***	1.35(1.27–1.44) ***
Perceived social benefits	1.47(1.39–1.55) ***	1.44(1.36–1.52) ***	1.33(1.25–1.41) ***	1.27(1.19–1.35) ***
Collectivism	1.31(1.25–1.38) ***	1.27(1.20–1.33) ***	1.32(1.25–1.41) ***	1.25(1.18–1.33) ***
National pride	1.17(1.11–1.23) ***	1.15(1.09–1.21) ***	1.12(1.05–1.19) ***	1.09(1.02–1.16) **

Note: ORc = Crude odds ratio; ORa = Adjusted odds ratio. ** *p* < 0.01; *** *p* < 0.001. Adjusted models were adjusted for studied province, gender, ethnicity, faculty, grade and perceived risk of COVID-19 infection.

**Table 4:** Interaction effects (standardized scores).

	Behavioral Intention of Free COVID-19 Vaccination with Frequent Mild Side Effects			
	80% Efficacy		50% Efficacy	
	ORa (95% CI)	ORa (95% CI)	ORa (95% CI)	ORa (95% CI)
	Model 1a	Model 1b	Model 2a	Model 2b
Perceived personal benefits	1.12 (1.05–1.20) **	1.13 (1.05–1.21) ***	1.30 (1.20–1.42) ***	1.29 (1.18–1.41) ***
Perceived social benefits	1.33 (1.24–1.43) ***	1.33 (1.24–1.43) ***	1.06 (0.97–1.15)	1.06 (0.97–1.16)
Perceived personal benefits × perceived social benefits		0.99 (0.95–1.03)		1.02 (0.98–1.06)
	Model 1c	Model 1d	Model 2c	Model 2d
Perceived personal benefits	1.31 (1.24–1.38) ***	1.31 (1.24–1.38) ***	1.30 (1.22–1.39) ***	1.29 (1.21–1.38) ***
Collectivism	1.20 (1.14–1.27) ***	1.21 (1.14–1.27) ***	1.19 (1.12–1.26) ***	1.17 (1.10–1.25) ***
Perceived personal benefits × collectivism		0.98 (0.94–1.02)		1.04 (1.00–1.09)
	Model 1e	Model 1f	Model 2e	Model 2f
Perceived personal benefits	1.34 (1.27–1.42) ***	1.35 (1.28–1.42) ***	1.34 (1.25–1.42) ***	1.34 (1.26–1.43) ***
National pride	1.09 (1.03–1.15) **	1.08 (1.03–1.14) **	1.03 (0.96–1.09)	1.03 (0.96–1.09)
Perceived personal benefits × national pride		0.95 (0.91–1.00) *		0.97 (0.92–1.02)

Notes: * *p* < 0.05; ** *p* < 0.01; *** *p* < 0.001. The models were adjusted for studied province, sex, ethnicity, faculty, grade, and perceived risk.

## Data Availability

The data was available on reasonable request.
